# Supplementary material for: Barking up the right tree: Univariate and multivariate fMRI analyses of homonym comprehension
Source: Neuroimage. 2020 Oct 1;219:117050. doi: 10.1016/j.neuroimage.2020.117050 (PMC7443701; doi:10.1016/j.neuroimage.2020.117050)
Supplement: Multimedia component 1 [file mmc1.docx]

Supplementary Materials

*Supplementary Table 1: Peak activation co-ordinates for univariate contrasts of task*

|  |  |  |  | MNI co-ords | | |
| --- | --- | --- | --- | --- | --- | --- |
| Effect | Location | Extent (mm^3^) | *t* | x | y | z |
| Semantic > Phonological | L ATL & IFG | 33880 |  |  |  |  |
|  | L anterior STS |  | 9.54 | -58 | -2 | -12 |
|  | L temporal pole/IFG |  | 8.64 | -44 | 20 | -14 |
|  | L pars orbitalis |  | 8.25 | -50 | 34 | -12 |
|  | R IFG & temporal pole | 12584 |  |  |  |  |
|  | R pars orbitalis |  | 7.68 | 36 | 34 | -14 |
|  | R temporal pole/IFG |  | 6.11 | 44 | 16 | -18 |
|  | R anterior STS |  | 5.49 | 52 | -4 | -22 |
|  | R cerebellum | 4648 |  |  |  |  |
|  | R cerebellum |  | 6.45 | 10 | -84 | -28 |
|  | R cerebellum |  | 6.38 | 12 | -80 | -36 |
|  | R cerebellum |  | 3.58 | 18 | -92 | -42 |
|  | L inferior parietal lobe | 8672 |  |  |  |  |
|  | L intraparietal sulcus |  | 6.22 | -32 | -78 | 48 |
|  | L angular gyrus |  | 6.04 | -46 | -78 | 26 |
|  | L temporoparietal junction |  | 4.74 | -56 | -66 | 24 |
|  | Ventromedial PFC | 5784 |  |  |  |  |
|  | Gyrus rectus |  | 5.90 | -4 | 50 | -14 |
|  | Gyrus rectus |  | 5.26 | -6 | 36 | -18 |
|  | Subgenual cingulate |  | 4.20 | -2 | 20 | -12 |
|  | Dorsomedial & L dorsolateral PFC | 22952 |  |  |  |  |
|  | Pre-supplementary motor area |  | 5.76 | -4 | 30 | 56 |
|  | Superior frontal gyrus |  | 5.60 | 0 | 42 | 42 |
|  | Pre-supplementary motor area |  | 5.25 | -4 | 28 | 66 |
| Phonological > Semantic | L inferior & superior parietal lobe | 34824 |  |  |  |  |
|  | L intraparietal sulcus |  | 10.87 | -48 | -40 | 46 |
|  | L superior parietal lobule |  | 6.68 | -22 | -64 | 52 |
|  | L intraparietal sulcus |  | 6.07 | -44 | -48 | 62 |
|  | L dorsolateral PFC | 8304 |  |  |  |  |
|  | L middle frontal gyrus |  | 7.30 | -44 | 40 | 20 |
|  | L middle frontal gyrus |  | 3.56 | -30 | 34 | 32 |
|  | L middle frontal gyrus |  | 3.29 | -30 | 48 | 32 |
|  | Medial PFC & L precentral gyrus | 24320 |  |  |  |  |
|  | Supplementary motor area |  | 7.29 | -4 | 4 | 68 |
|  | Precentral gyrus |  | 6.92 | -2 | -18 | 64 |
|  | L superior frontal gyrus |  | 6.65 | -22 | 2 | 52 |
|  | R inferior parietal lobe | 18064 |  |  |  |  |
|  | R postcentral gyrus |  | 6.83 | 48 | -30 | 52 |
|  | R intraparietal sulcus |  | 5.29 | 42 | -44 | 56 |
|  | R occipital cortex |  | 3.98 | 28 | -64 | 40 |
|  | R cerebellum | 5560 |  |  |  |  |
|  | R cerebellum |  | 5.59 | 28 | -62 | -52 |
|  | R cerebellum |  | 3.97 | 12 | -78 | -52 |
|  | R cerebellum |  | 3.33 | 32 | -46 | -50 |
|  | R occipitotemporal & cerebellum | 5664 |  |  |  |  |
|  | R posterior fusiform gyrus |  | 5.35 | 30 | -68 | -22 |
|  | R lingual gyrus |  | 4.83 | 8 | -58 | -6 |
|  | R lingual gyrus |  | 3.97 | 12 | -60 | 4 |
|  | R dorsolateral PFC | 5544 |  |  |  |  |
|  | R middle frontal gyrus |  | 5.03 | 42 | 42 | 16 |
|  | R middle frontal gyrus |  | 3.47 | 36 | 40 | 26 |
|  | R frontal white matter |  | 3.39 | 24 | 40 | 24 |

*ATL = anterior temporal lobe; IFG = inferior frontal gyrus; STS = superior temporal sulcus; PFC = prefrontal cortex.Supplementary Table 2: Peak activation co-ordinates for univariate contrasts in the semantic task*

|  |  |  |  | MNI co-ords | | |
| --- | --- | --- | --- | --- | --- | --- |
| Effect | Location | Extent (mm^3^) | *t* | x | y | z |
| Homonym > Unambiguous | L lateral PFC | 23384 |  |  |  |  |
|  | L pars triangularis |  | 6.56 | -40 | 32 | 14 |
|  | L pars triangularis |  | 6.01 | -38 | 14 | 30 |
|  | L pars opercularis |  | 5.90 | -34 | 8 | 24 |
|  | L intraparietal sulcus | 7408 |  |  |  |  |
|  | L intraparietal sulcus |  | 5.20 | -30 | -58 | 48 |
|  | L middle occipital gyrus |  | 4.97 | -30 | -70 | 42 |
|  | L intraparietal sulcus |  | 4.93 | -44 | -44 | 52 |
|  | Bilateral cerebellum | 4496 |  |  |  |  |
|  | R cerebellum |  | 5.13 | 26 | -72 | -50 |
|  | L cerebellum |  | 4.33 | -16 | -66 | -28 |
|  | L cerebellum |  | 4.10 | 16 | -76 | -46 |
|  | Dorsomedial PFC | 4856 |  |  |  |  |
|  | Presupplementary motor area |  | 4.94 | -8 | 22 | 48 |
|  | Presupplementary motor area |  | 3.96 | -2 | 32 | 48 |
|  | L anterior cingulate |  | 3.76 | -12 | 28 | 34 |
|  | L posterior temporal | 7296 |  |  |  |  |
|  | L ITG |  | 4.69 | -46 | -52 | -12 |
|  | L ITG/MTG |  | 4.14 | -52 | -68 | -6 |
|  | L ITG |  | 4.11 | -56 | -62 | -14 |
| Unambiguous > Homonym | Rostromedial PFC | 5456 |  |  |  |  |
|  | L medial PFC |  | 4.43 | -6 | 56 | 0 |
|  | Frontal pole |  | 4.22 | -4 | 58 | 10 |
|  | Gyrus rectus |  | 3.82 | 0 | 52 | -10 |
| Related > Unrelated | R inferior parietal & occipital | 15096 |  |  |  |  |
|  | R supramarginal gyrus |  | 7.74 | 60 | -32 | 54 |
|  | R angular gyrus |  | 4.79 | 50 | -46 | 42 |
|  | R supramarginal gyrus |  | 4.63 | 50 | -38 | 32 |
|  | L inferior parietal lobe | 19776 |  |  |  |  |
|  | L supramarginal gyrus |  | 6.56 | -56 | -42 | 38 |
|  | L angular gyrus |  | 6.42 | -62 | -50 | 42 |
|  | L angular gyrus |  | 5.81 | -52 | -64 | 38 |
|  | Ventromedial PFC | 10832 |  |  |  |  |
|  | Paracingulate gyrus |  | 6.42 | 0 | 48 | 10 |
|  | L frontal pole |  | 5.23 | -12 | 58 | 4 |
|  | Paracingulate gyrus |  | 5.23 | 8 | 48 | 12 |
|  | L mid-lateral temporal lobe | 5000 |  |  |  |  |
|  | L middle temporal gyrus |  | 5.74 | -66 | -34 | -8 |
|  | L middle temporal gyrus |  | 5.29 | -58 | -26 | -18 |
|  | Posterior cingulate | 6008 |  |  |  |  |
|  | Precuneus |  | 4.78 | -2 | -44 | 48 |
|  | Posterior cingulate cortex |  | 4.64 | -4 | -18 | 38 |
|  | Posterior cingulate cortex |  | 4.30 | 0 | -30 | 44 |
| Unrelated > Related | L lateral PFC | 13728 |  |  |  |  |
|  | L pars triangularis |  | 7.24 | -42 | 28 | 10 |
|  | L precentral gyrus |  | 6.47 | -40 | -2 | 34 |
|  | L middle frontal gyrus |  | 5.94 | -56 | 30 | 26 |
|  | Dorsomedial PFC | 5848 |  |  |  |  |
|  | R anterior cingulate |  | 5.99 | 10 | 22 | 42 |
|  | L anterior cingulate |  | 5.94 | -10 | 14 | 46 |
|  | L posterior ventral temporal | 4856 |  |  |  |  |
|  | L fusiform gyrus |  | 5.15 | -40 | -36 | -20 |
|  | L inferior temporal gyrus |  | 4.69 | -46 | -48 | -14 |
|  | L fusiform gyrus |  | 3.90 | -30 | -34 | -22 |

*PFC = prefrontal cortex; MTG = middle temporal gyrus; ITG = inferior temporal gyrus.*

*
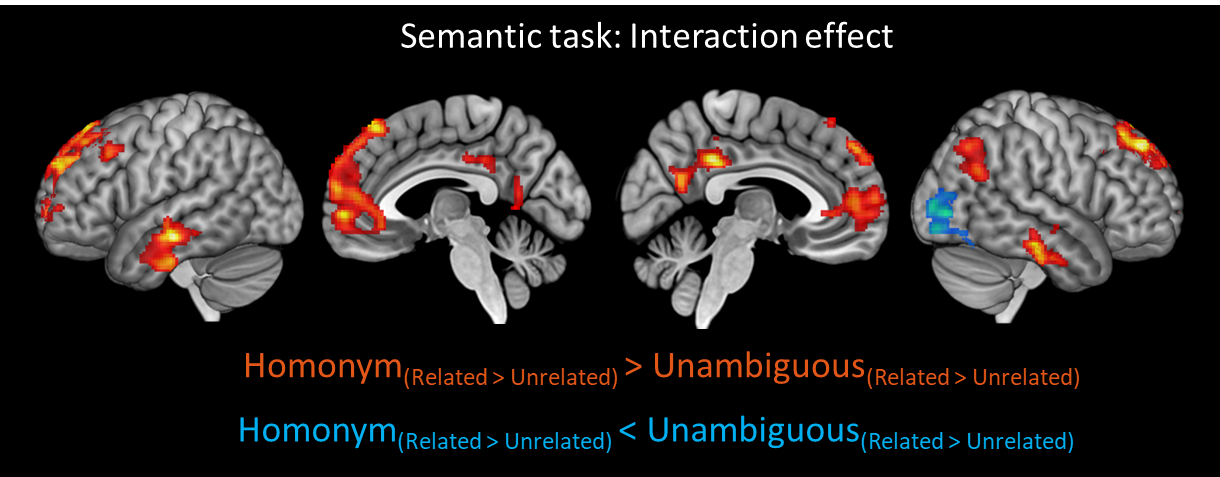
*

*Supplementary Figure 1: Whole-brain univariate activation contrasts for the interaction effect in the semantic task Images are shown at a voxelwise threshold of p<0.005, corrected for multiple comparisons at the cluster level.*

*
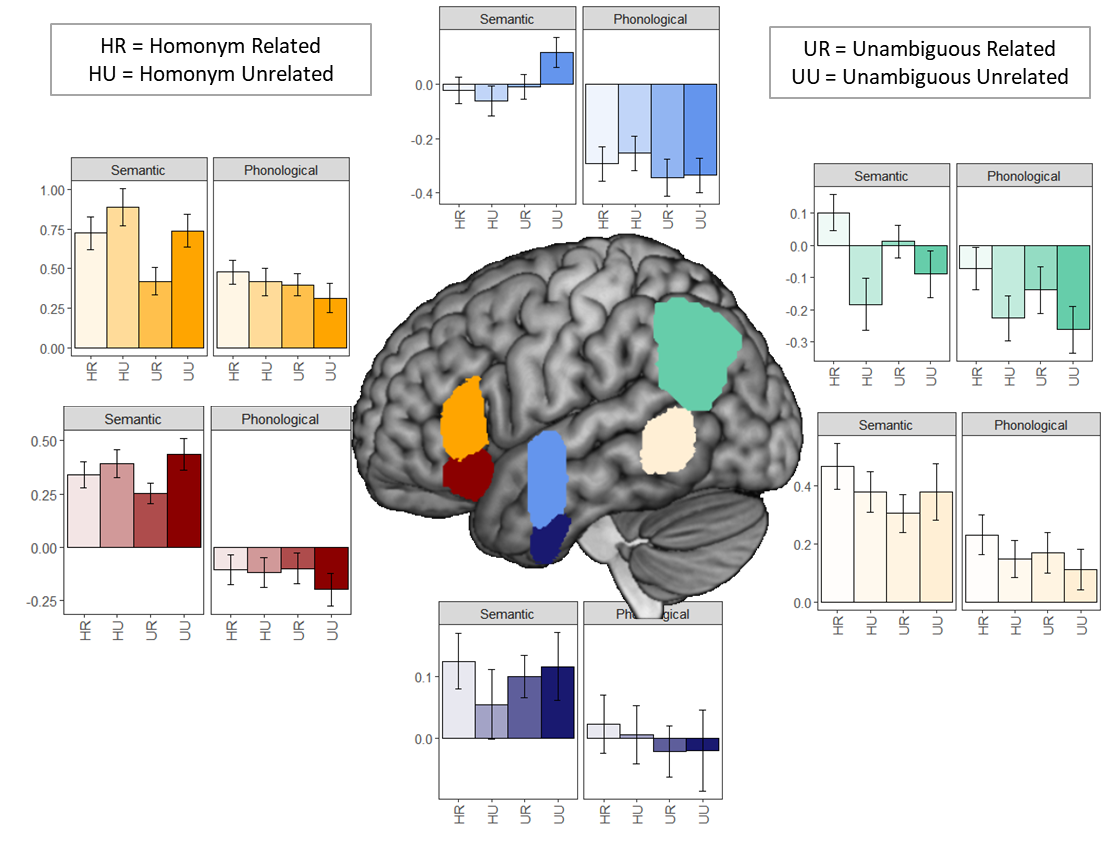
*

*Supplementary Figure 2: Activation in each region of interest as a function of condition Effect size is shown relative to implicit resting baseline. Bars indicate between-subjects SEM.*

*
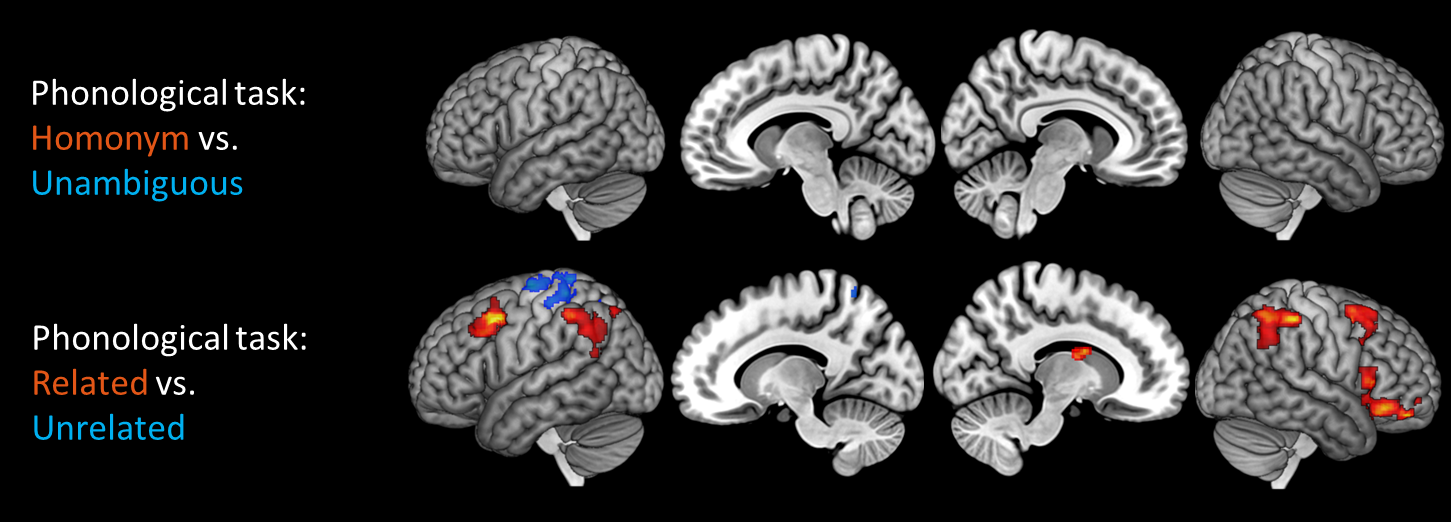
*

*Supplementary Figure 3: Whole-brain univariate activation contrasts for the phonological task Images are shown at a voxelwise threshold of p<0.005, corrected for multiple comparisons at the cluster level.*
